# Sea Buckthorn in Plant Based Diets. An Analytical Approach of Sea Buckthorn Fruits Composition: Nutritional Value, Applications, and Health Benefits

**DOI:** 10.3390/ijerph18178986

**Published:** 2021-08-26

**Authors:** Anca-Mihaela Gâtlan, Gheorghe Gutt

**Affiliations:** Food Engineering Faculty, “Ștefan cel Mare” University, 720229 Suceava, Romania; g.gutt@fia.usv.ro

**Keywords:** *Hippophaë* *rhamnoides*, sea buckthorn, plant-based diet, analytical characterization, nutritional value, applications, health beneficial properties

## Abstract

Current nutritional trends include plant-based diets as nutritional behavior of consumers who are increasingly concerned about a healthy lifestyle. Sea buckthorn (*Hippophaë* *rhamnoides* L.) is a plant with great virtues, containing more than 100 types of compounds. It is a plant with versatile properties, multiple economic advantages and a rich history, which still continues in natural medicine, and it is hence included in the daily diet by more and more people for the prevention and treatment of diet-related diseases. Its uniqueness is due to its chemical composition and the health beneficial properties that rise from its composition. This review is a detailed analytical picture of the current state of knowledge currently available regarding the *Hippophaë* plant, providing an overview of the qualities of sea buckthorn. This article summarizes data on sea buckthorn’s nutritional value, health beneficial properties, and its applications.

## 1. Introduction

The fruit of *Hippophaë* species is called a third-generation fruit [1]. This plant drew the attention of several researchers for centuries now, as it is a plant with versatile properties, with multiple economic advantages and it has been used in daily life for a variety of purposes ranging from raw material for obtaining food products, cosmetics, and nutraceutical preparations, but also for environmental protection. Moreover, *Hippophaë* plant has a rich history, which still continues, in natural medicine.

Sea buckthorn (*Hippophaë* L., family *Eleagnaceae*) is widely distributed in Asia, from seashores to mountainous areas, and North-Western Europe [2]. Approximately 150 species, subspecies, and varieties of sea buckthorn have been identified within Eurasia; they differed in the habitat of the shrub, the appearance of berries and their use-value [3]. Among them, *Hippophaë rhamnoides* is the most important and wide-spread in Europe [4]. Sea buckthorn is a dioecious and anemophilous plant, the pollination of female blossoms is possible by wind [2]. It withstands well on poor soils and is able to tolerate extreme temperatures ranging from −40 °C to +40 °C [5]. Since it has low requirements on growing conditions, it behaves invasively if it grows in low humid, alluvial gravel, wet landslips, and riverside [6,7]. Crop productivity is about 4–5 t per ha [3], in some cases 20–25 t per ha [2]. The harvest method usually applied is cutting shoots [2]. After harvesting, the shoots with berries undergo freezing at −38 °C, berries are separated from shoots without damage, then they are collected and processed [2]. Ripe berries are oval shaped and most often yellow, orange or red in colour, depending on the variety [8]. Berries, leaves, and bark are rich in many bioactive substances valuable for nutritional and health-promoting properties. From the berries, the two most common products are derived: juice from the fleshy tissue of berries, and oil produced from the seeds of berries [3]. The various nutrients and bioactive components present in all parts of Hippophae plant include minerals, vitamins, polysaccharides, unsaturated fatty acid, terpenoids, polyphenolic compounds, nonsteroidal compounds, flavonoids, organic acids, and volatile components [1,9]. Due to this exceptional chemical composition, sea buckthorn has a wide range of various positive biological, physiological, and medicinal effects, which were extensively described, such as antioxidative and immunomodulating, cardioprotective and antiatherogenic, antibacterial and antiviral effects, healing effect on acute and chronic wounds, antiradiation, anti-inflammatory, antidiabetic, anticarcinogenic, hepatoprotective, and dermatological effects, etc. [3,10,11,12,13,14,15].

The aim of this study is to offer a complete state of the art regarding the knowledge about sea buckthorn and what makes it a unique plant, starting from the earliest scientific concerns, to the latest discoveries regarding its chemical composition, therapeutic valences and areas of importance in which it is used. This analytical characterization of sea buckthorn will be of great use for the nutrition industry as a guide for the selection of food products for consumers who opt for a healthy lifestyle through a plant-based diet.

## 2. Sea Buckthorn in the Acceptance of Scientific and Popular Language

The scientific name of sea buckthorn, *Hippophaë rhamnoides* L., is commensurate with its qualities. The explanation for the etymology of this name differs from author to author. In the book “Sea buckthorn: A pharmacy in a plant”, the authors, Brad et al. [16] explain the consideration according to which the name refers to the use of sea buckthorn fruits in antiquity for the elimination of intestinal worms in horses. The explanation would be that the genus *Hippophaë*, as botanists called it, derives from the Greek terms hippos—horse and phao—I kill [16]. On the other hand, Olas [10] issued the information according to which an explanation of the name of *Hippophaë* would be hippo—horse and phao—light, which generally led to the translation “shiny horse hair”, which makes the whole bush “a shiny horse thorn” [10].

In fact, sea buckthorn is known in different parts of the world under various popular names, which are generally related to the area, always capturing the place, the presence of thorns, the color of the fruit or their effect on humans. In the Nordic countries, sea buckthorn is appreciated as the Oil Tree of the North, in Germany it is called sanddorn, and as popular names it appears under the terms of: sand willow, sea thorn, shore thorn, Rhine thorn, Haff thorn, grazing land thorn, meadow thorn, painful thorn, fire thorn, coral bush, red thorn, or pheasant fruit. The Dutch drastically called them “laxative fruits” because the acids in the fruit stimulate the intestinal transit. Russian speech includes 23 different names in the dialect for sea buckthorn. The name of the city in Uzbekistan (Dzbidda) means sea buckthorn. Other names, such as the thorny shrub or the willow bush, are related to its botanical characteristics. The Russian name Oblepicha means that the fruits are tightly attached to the branches. The English name sea buckthorn (the thorn of sea goat) is a bit more original—fans of natural sea buckthorn keep it that way, being against the association with backgeruch (goat smell). In Romania, it is known as: sea buckthorn, river white sea buckthorn, river sea buckthorn, spiny sea buckthorn, blue sea buckthorn, dracila, red sea buckthorn, and in Buzau county, in Cătina (Sea buckthorn) locality, it is called “the fruits of the Mother of God” [17].

## 3. Documentary Attestations of Sea Buckthorn

The first peoples to discover the therapeutic valences of sea buckthorn were the Thracians, Hindus, Greeks, Chinese, Mongols, Celts, and Slavs. The history of sea buckthorn dates back to traditional ethnobotanical and ethnopharmacological utilization of plant species, which was first documented in ancient Greek texts by Theophrastus and Dioscorides, in Ayurveda (classic ancient Indian system of medicine written in the period 5000–500 BC), who recommended feeding the race horses with sea buckthorn in order to increase their muscle mass [16]. The ancient Greeks began to use the individual elements of sea buckthorn for a variety of purposes. The young shoots and leaves were used as animal fodder, resulting in fast weight gain and visible improvements in hair quality, which became healthier and brighter, especially in horses [18].

Classic Tibetan medicinal literature, including the RGyud Bzi (four books of Pharmacopeia) dated to the times of Tang Dynasty (618–907 AD) also includes the utilization patterns of sea buckthorn, which explicitly recommended the consumption of sea buckthorn (star-bu) for those who traveled at high altitudes. In the 8th century AD, in the work “Djud-shi”, written by the famous physician Yuthog Yontan Gonpo, more than 300 medicinal preparations of sea buckthorn are presented, alone or in combination with other plants, minerals or even foods, processed as juice or as extracts, in the form of powders or pills, as lard or liqueur, in the form of patches, compresses, ointments, and pastes [17,19,20].

In the 13th century, the Mongolian pharmacologist Losang Que-Pei laid the foundations of a 120-chapter work synthesized from Tibetan medicine, which also contains many recipes based on sea buckthorn, among others for the treatment of diseases of the lungs, stomach, intestines, liver, gallstones, female diseases, rheumatic pains, and edema of the joints [16]. In “Dsejchar Migczan”, a reference book of ancient Tibet, of healing plants, which was discovered in the 19th century, in a history of Mongolian medicine, the action of sea buckthorn is described synthetically. Sea buckthorn has a hardening and velvety taste and it acts through the “bagdan”, healing through the lungs and throat. That is why the concentrate is used as the so-called “blood of the king’s heart”. Seeds can also be used, which break down evil, thin the blood, and heal the “bagdan”. Khanda—the thick extract of sea buckthorn oil, was used not only to regulate mucus, but also to heal so-called blood swellings, such as hematomas and hemorrhages. It helps in the treatment of inflammatory and infectious processes, it is mentioned especially as a stimulator of mucosal activity through the beneficial action on esophageal tumors, stomach and bleeding inflammation of the appendix [19].

The plant’s virtues have not gone unnoticed even in modern times, when they were first used experimentally in the diet of Soviet cosmonauts because, in addition to the other qualities of sea buckthorn, Moscow scientists have found that it protects the human body from cosmic radiation. The potential of sea buckthorn led to its introduction as a crop plant in the USA, Canada, Germany, Russia, China, and Ukraine. In Europe and Central Asia, people used every part of the plant, the fruits, leaves, roots or bark, as a food, dietary supplement, firewood, fuel, feed, a decorative element, or even to fence their property [20].

In Romania, the first research actions in the field of sea buckthorn regarding the “obtaining of vitaminizing concentrates and the superior capitalization of sea buckthorn” started in 1953, at the initiative of the university professor Ion Brad. He was the first researcher in Romania to study the biochemical composition of fruits, conducted scientific studies on sea buckthorn preparations and their use in medicine, food, cosmetics, and animal feed. The first doctor in sea buckthorn in Romania is Emanoil Grigorescu (1963), with the thesis “Contributions to the pharmacognostic and phytochemical study of indigenous *Hippophaë rhamnoides* L.”, initially coordinated by prof. dr. Elemer Kopp and then by prof. dr. Teodor Goina. The promoter of sea buckthorn culture in Romania is prof. dr. eng. Victor Cireașă, the first to notice that the plant can be taken from the spontaneous flora and introduced into the culture, referring in many times to the sea buckthorn fruit as “the strongest fruit with divine oil”, the medicinal berries of the Mother of God” [17].

Worldwide, in 2001, the International Sea buckthorn Association (ISA) was founded, the first international institution for the promotion and development of sea buckthorn. The first Sea Buckthorn Congress took place from 14 to 18 September 2003 in Berlin, Germany and was organized by the German Sea Buckthorn Association, Humboldt University, and Technical University Berlin.

## 4. The Nutritional Value of Sea Buckthorn

Research conducted worldwide has shown that sea buckthorn leaves, fruits, and shoots contain a number of biologically active substances with an essential role in regulating metabolism [17]. Sea buckthorn fruits are among the most nutritionally complex of all berries [21]. Berries are rich in many essential nutrients such as polyunsaturated fatty acids, provitamins A, C, E, and a wide variety of bioactive compounds. The mesocarp of the fruit contains high levels of carotenoids, phenolic compounds, free and esterified sterols, triterpenols and isoprenols [22].

The phytochemical and nutritional composition of sea buckthorn berries differs considerably depending on the species, components analyzed, climatic and growing conditions, variations between years, degree of maturation, storage conditions, time of harvest, and method of processing and analysis (Table 1) [23].

### 4.1. Major Nutrients

#### 4.1.1. Lipids and Fatty Acids

Morphologically, sea buckthorn berries consist of seeds (23% *w*/*w*), pulp (68% *w*/*w*), and skin (8% *w*/*w*) [27]. Sea buckthorn fruits are distinguished by the fact that they contain oil in considerable quantities, as an inherent part of the fruit [28]. The most valuable component of sea buckthorn fruits is their oil [29]. There are two sources of oil in sea buckthorn fruit: seed oil and oil contained in the pulp of the fruit, quantitatively more compared to seed oil [2]. 

In general, the oil in the pulp/peel fraction is combined due to separation difficulties. Both seed oil and pulp oil have high total lipid content. The composition of sea buckthorn seed and pulp oils varies depending on the subspecies, origin, crop care activities, fruit harvesting time, and extraction method [29]. Dulf F. [28] reported the oil content of whole grains, pulp and seeds (taking into account fresh weight) of different sea buckthorn varieties in Romania (*carpatica* ssp.): 45–84 g/kg in whole grains, 45–88 g/kg in the pulp, and 106–135 g/kg in the seeds. The yield of oil extraction differs depending on the drying method of the component parts of the fruit: 36% for sea buckthorn pulp dried in air stream, compared to 16% for lyophilized pulp (*w*/*w*), while for seeds the values were similar: 11% and 12%, respectively (*w*/*w*) [28].

The main constituents of neutral lipids in sea buckthorn are cerides (esters of C20–C26 fatty acids with aliphatic alcohols), concentrated in the peel of the fruit and in the seed, C23–C29 hydrocarbons (most of the surface layer of the peel) and phytosterols (70–100% β-sitosterol, α- and β-amyrins, erythrodiol and other constituents of the unsaponifiable lipid fraction of seeds) [29].

Sea buckthorn oil contains, on average, 35% palmitoleic acid (16:1n-7), a rare and valuable acid, a component of skin fat, being known for its ability to support cell tissue and speed wound healing [30], as well as for hypocholesterolemic and hypoglyceridemic actions [31]. Sea buckthorn seed oil is characterized by a high content of oleic acid (17%) and a one-to-one ratio of omega-3 (alpha linolenic) and omega-6 (linoleic) at about 34% and 31%, respectively. The equivalence relationship between the two omegas is particularly important, intervening in the regulation of thousands of metabolic functions. Almost every biological function is interconnected with the balance between omega-6 and omega-3 [30,32].

Seed oil and sea buckthorn pulp oil differ considerably in the composition of fatty acids. Saturated fatty acids are found in the oil extracted from the pulp, mainly palmitic acid, while the seed oil contains C18 type unsaturated fatty acids, such as linoleic acid (18:2) and linolenic acid (18:3) [33,34]. In sea buckthorn pulp oil, Europe ssp., the main fatty acids identified were palmitoleic acid (16:1n-7), palmitic acid (16:0), and oleic acid [30].

Dulf F. [28] studied the fatty acid composition of oils from the pulp/peel, seeds and whole fruits of six sea buckthorn varieties in Romania (*Carpatica* ssp.) and also the oil content of the seeds, pulp/peel and whole sea buckthorn fruits (based on fresh weight) of different varieties (*Carpatica* ssp.). The oil quantities from different parts of sea buckthorn fruit varied as follows: 45–84 g oil/kg whole berries, 45–88 g oil/kg sea buckthorn pulp/peel, 106–135 g oil/kg sea buckthorn seeds. Due to the predominance of pulp and peel in sea buckthorn fruit, the composition of whole fruit oil is similar to that of pulp/peel oil. The levels of fatty acids in seed oil and in sea buckthorn peel varied greatly between the six studied varieties [28]. The predominant fatty acids in the oils from sea buckthorn pulp/peel were palmitic (23–40%), oleic (20–53%), and palmitoleic (11–27%). Small amounts or traces of vaccenic acid, linoleic acid, α-linolenic acid, stearic acid, myristic acid, pentadecanoic acid, cis-7 hexadecanoic acid, margaric acid, and two long chain fatty acids, arachidic acid and eicosanoic acid were detected in all oils from the soft parts of the sea buckthorn fruit. Monounsaturated fatty acids are the most important class of fatty acids in terms of quantity (53–70%), followed by saturated fatty acids (26–41%), and then polyunsaturated fatty acids (3–7%) [28].

Similar amounts of palmitic acid (in *Indian-summer* variety and, *H. rhamnoides* (India)), vaccenic acid (in *Indian-summer* variety and *sinensis* subspecies) and α-linolenic acid (in *Indian-summer* variety, *H. rhamnoides* (India) and *H. salcifolia*) have been reported by various authors for sea buckthorn pulp oils. Higher proportions of palmitoleic acid and much lower levels of oleic acid are characteristic for sea buckthorn pulp oils from subspecies in Finland, China, and Canada, except for *H. tibetana* species, which had similar percentages of oleic acid compared to *H. carpatica* [4,33,35].

Sea buckthorn seed oils contain mainly the following acids: linoleic, α-linolenic, oleic, palmitic, and stearic, with small amounts or traces of vaccenic, palmitoleic, arachidic, eicosanoic, myristic, pentadecanoic, and margaric acid. A notable feature of sea buckthorn seed oils is the extremely low level of palmitoleic acid (0.1–0.5%). Large relative deviations were observed for oleic acid (13–21%) and linoleic acid (33–43%) concentrations. Unlike pulp oils, seed oils had higher amounts of polyunsaturated fatty acids (65–72%) and lower amounts of monounsaturated fatty acids (16–21.5%) and saturated fatty acids (11–16%), respectively [28].

The high content of palmitoleic acid, unusual for a vegetable oil, distinguishes the oils from sea buckthorn pulp/peel from those from sea buckthorn seeds [31]. Palmitoleic fatty acid forms a large part of epidermal lipids of human skin and therefore the pulp oil of sea buckthorn is often used in cosmetics emulsion. Many studies on palmitoleic acid have focused on the health of the skin and mucous membranes, based on the fact that omega-7 is present in both body structures [29,36,37]. 

Additionally, sea buckthorn is a very good source of phytosterols, which play an important role in the prophylaxis of cardiovascular diseases induced by hypercholesterolemia. Sitosterol has recently been intensively investigated for its physiologically beneficial effects on humans, being associated with reduction of cancer incidence [3]. Phytosterols are the main constituents of the unsaponifiable fraction of sea buckthorn oils [2]. The sterol content in sea buckthorn berries is from 2.2 to 8.8%. At least 17 types of sterols have been identified in the sea buckthorn, of which β-sitosterol is quantitatively the most important (Table 2). The total sterol content in seed oil varies from 12 g/kg to 23 g/kg, in soft parts between 10 g/kg and 29 g/kg and in the whole fruit the variation range is between 13 g/kg and 33 g/kg. Sitosterol represents 57–76% of total sterols in seeds and 61–83% of total sterols in soft parts [3]. The most important phytosterols in seeds are δ-5-Avenasterol and obtusifoliol (approx. 15–17% of total sterols) and together with stigmasta-8-ene-3β-ol are about 5–6 and 8–10% of total sterols from soft parts and whole fruits, respectively [2,38]. Sea buckthorn is a better source of β-sitosterol than other very popular oils such as sunflower oil or virgin olive oil [3], and the total amount of phytosterols in sea buckthorn exceeds 4 to 20 times the amount in soybean oil [2]. Therefore, sea buckthorn is obviously worthy of being considered as a source of phytosterols in the diet.

#### 4.1.2. Carbohydrates and Fibers

One of the main components in sea buckthorn fruit dry matter is the carbohydrates class. Recent researches have reported total carbohydrates content in the range of 400 and 600 g/kg dry weight [35,39].

The soluble sugar content determined refractometrically varies from 9.3 to 22.74°Brix for sea buckthorn juice [2]. This variation, over a wide range of values, is due to the time of harvesting the fruits, respectively, their degree of ripeness. They can be harvested in autumn, at full maturity, or in winter, after the fruit freezes [36]. In addition, the sugar content varies depending on the origin, population, and genetic background of the plant [37].

The sugar content in sea buckthorn berries varies in the range 27–58 g/kg DW and increases during ripening of the berries [19]. A total of 90% of sugars in Chinese and Russian varieties are glucose and fructose, as against the Finnish varieties where glucose and fructose sum 60% of sugars. When comparing subspecies, the *sinensis* one was found to have the highest sugars content in pulp juice, up to a level of 105 ± 36 g/L [36].

Generally speaking, the carbohydrates contained in sea buckthorn berries are mostly glucose and fructose, with small amounts of xylose, mannitol, sorbitol, xylitol [37], saccharose, and ethyl glucose [40]. Yang et al. [41] determined a methyl inositol, in addition to ethyl glucose, a unique saccharide derivative in sea buckthorn berries. Moreover, cyclitol is a functional component because of its potential role in scavenging hydroxyl radicals [41].

Sugars in conjunction with organic acids may effectively influence the sensory properties of sea buckthorn berries, which play an important role in market acceptance by consumers [37]. As sea buckthorn berries have a low sugar content and a high titratable acidity, an important factor in improving the flavor of sea buckthorn berries is a high sugar/acid ratio [42], which varies by changes in latitude and altitude of geographic location [43].

In addition, one of the important health-promoting aspects of sea buckthorn is high fiber content, which varies depending on weather conditions and maturity of the berries. The level of crude fiber in sea buckthorn is between 62 g/kg dry weight [26] and 100 g/kg dry weight [35]. Jaroszewska et al. [26] reported that sea buckthorn has an abundant content of dietary fibers. The dietary fibers fractions in sea buckthorn berries are distributed as follows: 160–200 g/kg dry weight neutral detergent fiber, 120–145 g/kg dry weight acid detergent fiber, 50–70 g/kg dry weight acid detergent lignin, 45–55 g/kg dry weight hemicellulose, and 60–75 g/kg dry weight cellulose [26]. 

The polysaccharides of sea buckthorn berries are an especially non-starchy type of polysaccharides, composed of cellulose, hemicelluloses, pectin, and hydrocolloids that are together with lignin the major constituents of dietary fiber [3]. Only sea buckthorn seeds contain starch, a dietary polysaccharide with high digestibility in the human small intestine, in an amount of 49 g/kg dry weight. Both peel and pulp contain pectin, in much smaller quantities of 5 g/kg dry weight and 15 g/kg dry weight, respectively. The content of raw fiber in seeds was 130 g/kg dry weight, in peel 66 g/kg dry weight, and in pulp 47 g/kg dry weight [44]. 

#### 4.1.3. Proteins and Amino Acids

Various parts of sea buckthorn plant (woody verdure, seeds, leaves, barks, branches) have a high protein content [3]. The most considerable amounts of protein (on average 15%) are found in sea buckthorn leaves, and for this reason they are used as an unconventional source of protein in human food [45].

Distribution of protein in sea buckthorn berry varies widely in particular parts, the sea buckthorn seeds being considered a unique protein source [3]. In Mongolian wild sea buckthorn species, approximately 38% of total protein was found in seeds while seeds represented 7.2% of fresh berries [46]. 

Comparing to other berry varieties, the berries of sea buckthorn are characterized by a relatively high content of protein [18]. In addition, the protein levels in sea buckthorn juice are quite high for a fruit juice and this is reflected in the fact that sea buckthorn juice is a cloudy or opalescent product. The source of opalescence in most juices is due to the presence of cellular debris, but largely due to the presence of cell membranes that contain considerable proteins and give a stable turbidity to the juice [2]. The total protein content reported for various species of sea buckthorn were 46–129 g/kg dry weight (India variety) [35] and 93 g/kg dry weight (Polish variety) [26]. 

Sea buckthorn juice is rich in many free amino acids. A total of 18 of the 22 known amino acids have been found in sea buckthorn fruits, half of which are essential because they play a critical role in various processes in the human body [47,48]. Of these, eight free amino acids (threonine, valine, methionine, leucine, lysine, tryptophan, isoleucine, and phenylalanine) are essential for the human body [30,45]. Leucine and lysine presented in sea buckthorn are deficient in the majority of other plant feedstuffs. Methionine and cysteine were observed as the limiting amino acids [45].

Zhang et al. [48] reported aspartic acid as the predominant free amino acid in sea buckthorn berries (426.6 mg/100 g), followed by proline (45.2 mg/100 g), and threonine (36.8 mg/100 g) (Table 3) [48], while Stobdan et al. [45] reported a different hierarchy in terms of quantity, the dominant amino acid in sea buckthorn being asparagine, followed by glutamic acid and alanine [45]. Asparagine was also found quantitatively predominant in sea buckthorn in other more recent studies [10,49]. Since the full range of proteinogenic amino acids and essential amino acid content in sea buckthorn is high, it is within a class of relatively high-quality plant protein resources [10].

#### 4.1.4. Organic Acids

Sea buckthorn fruits contain organic acids, mainly malic acid and quinic acid, which together make up about 90% of all fruit acids of different origins. Large variations have been reported between the concentrations of sea buckthorn acids of different origins. The subspecies of *Hippophaë rhamnoides* L. from Russia showed relatively low concentrations of total acidity (2.1–3.2 g/100 mL), the Finnish genotypes were at an intermediate level with a range between 4.2 and 6.5 g/100 mL, while Chinese genotypes showed the highest concentrations of organic acids, with values between 3.5 and 9.1 g/100 mL [30,48]. Depending on fruits’ origin, Tang X. [52] reported variations of malic acid content in sea buckthorn juice between 11 and 60 g/L, quinic acid content between 7 and 49 g/L, 0.2–0.6% of all acids succinic acid, 0.04–0.3% of all acids citric acid and 0.013–0.014% of all acids tartaric acid [52].

#### 4.1.5. Mineral Elements

Sea buckthorn is recommended as a good source of essential mineral elements [3]. The study “Food Composition and Nutrition Tables” [51] revealed that the ash content in sea buckthorn berries is 4500 mg/kg. Because of the high content of water (82.6%), the ash content corresponds to 2.59% on dry weight basis. Alkali metals and alkali earth metals contribute considerably to the total mineral content [51]. 

There are many elements and trace elements in sea buckthorn, as listed in Table 4 [48,50,51,53,54]. Potassium is the most abundant of all the trace elements identified [55]. The contents of four main metals and phosphorus decrease in order potassium > calcium > phosphorus > magnesium ≈ sodium [51] (Table 4). However, the published data concerning sea buckthorn samples originated from different countries are quite inconsistent in the reported concentrations of individual elements. It is natural that the content of an element in plant material depends on many factors including variety or species, the part of plant, an area of cultivation, a composition of soil, an application of fertilizers, a degree of maturity, etc. All these factors manifest themselves also in the case of sea buckthorn samples of different origin [3].

Considering all data and comparing the trace element contents in other berries such as raspberries, blueberries and black currant, sea buckthorn contains less manganese and iron. Although the total iron concentration in sea buckthorn is low, its biological accessibility is most likely good because of the positive effect of high ascorbic acid content on iron absorption [15]. Besides that, the high selenium content is noteworthy, known for its antioxidant properties and role in boosting immunity and fertility [56,57].

As far as the toxic elements are concerned, their content in sea buckthorn is very low. Data in our previous report of analyses of sea buckthorn berries and juice belonging to *Hippophaë rhamnoides* L. ssp. *carpatica* showed low contents of arsenic, lead, and cadmium, significantly below the maximum amount allowed by regulations in effect [56]. These data are consistent with those reported by Gutzeit et al. [54] who found that heavy metals contents were bellow detection limit in all studied samples [54]. 

### 4.2. Lipophilic Components

#### 4.2.1. Carotenoids

Various carotenoids are the main substances present in large quantities in the pulp of sea buckthorn fruits [33,58], acting as an antioxidant and helping to synthesize and epithelialize collagen [23].

It is known that the total amount and the occurrence of different types of carotenoids is highly variable depending of the genetic origin, conditions of cultivation, climate, and harvesting time [3]. Pop et al. [59] reported a total carotenoid content of a Romanian *H. rhamnoides* variety of 860 mg/kg dry weight of berries, identifying zeaxanthin dipalmitate and other zeaxanthin esters, β-carotene, non-conjugated zeaxanthin, lycopene and β-cryptoxanthin palmitate as dominant carotenoids [59,60]. Ursache et al. [25] reported 12 compounds of the carotenoid class when analyzing sea buckthorn fruits from ssp. *Carpatica*, as follows: astaxanthin, zeaxanthin, zeaxanthin palmitate, γ-carotene, cis β-carotene, β-cryptoxanthin, lycopene, myristo-lutein palmitate lutein di-palmitate, β-carotene, α-carotene, and zeaxanthin di-palmitate. Of these, zeaxanthin was by far quantitatively the most important of the identified carotenoids (81.29 mg/g dry weight), followed by β-carotene (15.19 mg/g dry weight), astaxanthin (11.94 mg/g dry weight), β-cryptoxanthin (8.93 mg/g dry weight), and lycopene (2.24 mg/g dry weight) [25]. On the other hand, Xiao-Hua et al. [61] reported a β-carotene content in sea buckthorn fruits of Chinese variety of 100 mg/kg fresh weight, higher than that of pumpkin and double that of carrots, and this concentration does not decrease after the fruit is frozen [61]. Additionally, Ranjith et al. [31] compared different species of sea buckthorn from Indian Himalayas regarding their carotenoids content. They reported that berries from *Hippophaë rhamnoides* produced the highest amounts of carotenoids compared to *H. tibetana* and *H. salicifolia* [31].

The carotenoid content is one of the key characteristics by which sea buckthorn oil is traded commercially. Carotenoids in oil vary widely depending on the source of the oil from 0.5 to 21.4 g/kg [2].

#### 4.2.2. Tocochromanols

Tocopherols and tocotrienols (named tocochromanols), commonly known as vitamin E, are important bioactive components in sea buckthorn berries, which have a significant antioxidative effect [33]. The level of tocochromanols in berries depends on origin, variety, harvesting time, and maturation. In comparison with other fruit and vegetable, sea buckthorn berries are a rich source of tocochromanols, especially α-tocopherol [3]. The content of vitamin E in sea buckthorn is higher than in the germs of wheat, saffron, corn, and soybeans [30], but better sources of α-tocopherol than sea buckthorn are sunflower seeds, almonds, and hazelnuts [62]. Kallio et al. [33] reported a total content of vitamin E in *H. rhamnoides* ssp. *sinensis* and ssp. *mongolica* in the ranges 56–140 mg/kg fresh weight and 1.5–8.1 mg/kg fresh weight, respectively, α-tocopherol being the dominant tocopherol (76–89% of total tocopherols) [33]. 

On the other hand, Ranard et al. [63] stated that both, seed and pulp sea buckthorn oils are rich in α- and γ-tocopherols (444–1550 mg/kg and 461–1349 mg/kg of seed oil and 630–1940 mg/kg of α-tocopherols in pulp oil) compared to other vegetable oils such as virgin olive oil (98–370 mg/kg), sunflower oil (432 and 92 mg/kg), corn oil (173 and 260 mg/kg), canola oil (120 and 122 mg/kg), and soybean oil (71 and 273 mg/kg) [63]. Besides that, other studies also reported a high content of total tocopherols in sea buckthorn oil: Xiao-Hua et al. [61] concluded that each 100 g of sea buckthorn oil contains 206.9 mg of vitamin E [61] and Zadernowski et al. [64] showed that the oil prepared from whole berries of H. rhamnoides cultivars contained 1014–1283 mg/kg of total tocopherols, in which α-tocopherol was 62–68% and δ-tocopherol 32–38% of total tocopherols [64]. 

### 4.3. Hydrophilic Components

#### 4.3.1. Ascorbic Acid

Ascorbic acid (vitamin C) is the most important therapeutic element in sea buckthorn fruit, as it acts as an antioxidant and supports the integrity of the cell membrane [23,65]. It has been found in practically all parts of the sea buckthorn plant: in berries juice (11.6–13.0 g/kg), in seeds (1.5 g/kg), and in leaves (up to 3.7 g/kg) [66].

In *H. rhamnoides*, extensive variations of vitamin C were found between different shrubs, populations, and subspecies. The concentration of vitamin C varies from 0.3 to 3.1 g/kg fruit in the European *rhamnoides* subspecies, from 0.4 to 3 g/kg fruit in Russian varieties belonging to the *mongolian* subspecies, from 4.6 to 13.3 g/kg fruit in the *fluviatilis* subspecies and from 2 to 25 g/kg fruit in the Chinese *sinensis* subspecies [55]. 

The ascorbic acid content in sea buckthorn berries is 5 to 100 times higher than most other fruits or vegetable crops independently of *Hippophaë* species [3]. Thereby, the vitamin C content in sea buckthorn was found to be 20 times higher than that of hawthorn, 3 times higher than in kiwi, 6 times higher than in citrus, 80 times higher than in tomatoes, and 200 times higher than in apples [61].

It should be noted that sea buckthorn berries do not contain ascorbate oxidase, the enzyme responsible for the degradation of ascorbic acid and therefore, sea buckthorn products and even dried fruits still contain large amounts of vitamin C [67].

#### 4.3.2. Phenolic Compounds

The antioxidant activity of vegetal foods is mainly conditioned by their content in polyphenols. Phenolic compounds are the main compounds of the plant that have antioxidant activity. This activity is mainly due to the redox properties, which play an important role in the adsorption and neutralization of free radicals or the decomposition of peroxides [25].

The whole plant of sea buckthorn—berries, roots, leaves, stems and branches—contain various kinds of phenolics, including flavonoids, phenolic acids, and hydrolysable tannins [68]. Sea buckthorn extract has a total polyphenol content of 140.14 ± 6.64 mg GAE/g dry weight [25].

A total of 15 phenolic compounds, classified into 4 categories (phenolic acids, flavones, flavonol-monoglycosides, and flavonol-diglycosides), were identified by Guo et al. [11] only in the free fractions of all sea buckthorn subspecies, using the RP-HPLC technique. Of these, flavonol-diglycosides were predominant: 233 ± 46 mg/100 g dry weight, followed by flavonol-monoglycosides, phenolic acids and flavones (147 ± 24 mg/100 g dry weight, 62.9 ± 23.4 mg/100 g dry weight and 30.9 ± 5.5 mg/100 g dry weight, respectively) [11].

Flavonoids are the most common polyphenols found in food, especially their glycosides, which form the largest group of antioxidants found in nature [69]. Their concentration in sea buckthorn berries is several times higher than the content recorded in other high-flavonoid plants such as hawthorn, cornelian cherry, wild grown European blackberry, blackthorn or dog rose, mulberry, pomegranate, red raspberries, and blueberry [1,70]. Flavonol glycosides form the most concentrated class of phenolic compounds in sea buckthorn [20]. They are found mainly in glycosylated forms of the aglycones of isorhamnetin, quercetin, myricetin, and kaempferol [10,70]. The most abundant flavonoid compounds in sea buckthorn are isoramnetin glycosides and quercetin derivatives, the latter being the most significant in terms of quantity [20]. It has been shown that flavonol glycosides may play an important role in the prevention and management of chronic diseases such as cancer, diabetes, and cardiovascular disease [25]. In addition to the benefits to human health, the composition and flavonol glycosides content of sea buckthorn may influence the sensory perception of sea buckthorn products. Therefore, the composition and content of flavonol glycosides are among the most important indicators of the quality and therapeutic potential of sea buckthorn fruits [20].

Regarding the phenolic acid content of sea buckthorn fruit, Zadernowski et al. [71] described that the salicylic acid is the dominating phenolic acid in sea buckthorn berries. Its concentration ranged from 21 up to 47 mg/kg dry weight of berries depending on the variety, followed by p-coumaric acid (1.4–9.8 mg/kg dry weight), caffeic acid (concentration up to 6.7 mg/kg dry weight), gallic acid (1.0–4.6 mg/kg dry weight), and vanillic acid (0.5–1.8 mg/kg dry weight) [71]. However, other studies reported a different hierarchy of the identified phenolic acids in sea buckthorn. Bittova et al. [72] stated that gallic acid in free form is dominating, with a variable concentration occurring in leaves (79 mg/kg) and berries (16.9 mg/kg), while other phenolic acids such as caffeic acid, p-coumaric acid, and ferulic acid are found in a lower amount in sea buckthorn [72]. These data are partially in accordance with those reported later by Guo et al. [11] who found that gallic acid is the most important phenolic acid as quantity in sea buckthorn fruits originating from *sinensis* ssp., but the protocatechuic acid is quantitatively predominant in *yunnanensis* ssp., *mongolica* ssp. and *turkestanica* ssp. [11]. A more recent study, authored by Ji et al. [1] stated that gallic acid is the predominant phenolic acid in both the fruits and leaves of *Hippophaë* species [1].

In addition to flavonoids and phenolic acids, *Hippophaë* species contain tannins. Tannins are water-soluble polyphenol compounds found in alkaloids, polysaccharides, and proteins with relatively high molecular weight [1,73]. Tannins in sea buckthorn are divided into two groups: hydrolysable and condensed tannins. Gallo- and ellagitannins of monomeric type are the most abundant subgroups of hydrolysable tannins and includes stachyurin, casuarinin, casuarictin, hippophaenin B, strictinin, and isostrictinin [68,74]. Condensed tannins typically consist of two or more monomeric (+)-catechin or (-)-epichatechin units (procyanidins), while the other group consist of (+)-gallocatechin or (-)-epigallocatechin units (prodelphinidins) [75]. They are present in higher concentrations in sea buckthorn seeds, roots, flowers, green berries, and stems [3,76].

### 4.4. Aroma Compounds

There are very few researches in which the aroma compounds that are responsible for the sensory properties of sea buckhorn juices are studied [4,21,46,77,78,79].

Sea buckthorn fruits have a unique flavor that cannot be compared to the flavor of any other fruit [4]. As mentioned previously, the chemical composition of sea buckthorn is influenced by several factors, such as genotype, degree of maturity, weather conditions, growth location and latitude, and harvest date and, therefore, the sea buckthorn flavor is conditioned by these factors, too.

Sea buckthorn fruits are known to have a specific scent based on around 45 compounds, including esters, alcohols, aldehydes, ketones, and terpenes. Most subjects defined this smell with notes of berry or citrus, while Scandinavians defined it as pineapple scent [19]. Socaci et al. [80] identified 43 volatile compounds in sea buckthorn juice by GC—MS analysis. The most abundant derivatives were ethyl esters of 2-methylbutanoic acid, hexanoid acid, methylbutanoic acid, octanoic acid, and butanoic acid, as well as 3-methylbutyl 3-methylbutanoate, 3-methylbutyl 2-methylbutanoate, and benzoic acid ethyl ester [80]. On the other hand, Vitova et al. [81] identified 69 volatile compounds in sea buckthorn: 26 alcohols, 12 aldehydes, 11 ketones, 9 acids, and 11 esters [81].

The taste of sea buckthorn juice was characterized by astringency, sourness and bitterness, even when varying sugar concentration (from 3.7 to 45.8 g/kg berries) or titratable acidity (from 23.6 to 46.6 g citric acid/kg berries) [42]. Ma et al. [82] reported that ethyl-β-D-glucoside content, which is high in overripe sea buckthorn berries, is a negative factor for the pleasantness of the juice, contributing to the bitter flavor. The bitterness of the juice may be reduced by glucose [82]. The same study revealed that flavonol glycosides induce a silky, mouth-drying, and mouth-coating astringent sensation even at very low concentrations and that malic acid and isorhamnetin glycosides were the major compounds responsible for the astringency in sea buckthorn puree [82].

### 4.5. Bacterial and Fungal Microorganisms

Lukša J. et al. [77] studied the taxonomic profiles of bacterial and fungal microorganisms associated with sea buckthorn. In total, 6 bacterial phyla (35 families and 56 genera) and 4 fungal phyla (58 families and 108 genera) were identified. The dominant phylum of the entire population of prokaryotic microorganisms was Proteobacteria (71.1%), represented mainly by the classes Alphaproteobacteria and Gammaproteobacteria. Regarding the population of eukaryotic microorganisms, Ascomycota was found to be the most important group, representing 89.4% of the total number of sequences detected, followed by Basidiomycota (8.2%). The major group of operational taxonomic units in Ascomycota belonged to the class Dothideomycetes, while Basidiomycota was represented by members of the class Tremellomycetes [77].

At a higher taxonomic resolution, the bacterial community was predominantly dominated by the families Enterobacteriaceae (31.4%), Microbacteriaceae (16.3%), and Pseudomonadaceae (14.1%), represented by the most abundant genera Pantoea (16.8%), Okibacterium (14.4%) and Pseudomonas (14.1%). The community of fungal microorganisms was dominated by the families Dothioraceae (78%), followed by Davidiellaceae (2.4%) and traces of Taphrinaceae and Saccharomycodaceae. At the genus level, the vast majority of sea buckthorn associated fungal microorganisms (87.9%) have been described as unidentified [77].

### 4.6. Antioxidant Properties

Antioxidants are compounds that inhibit or delay the oxidation of other molecules by inhibiting the initiation or spread of chain oxidative reactions [78]. An antioxidant effect refers to enhancing the activity of antioxidant enzymes and inhibiting the activity of related oxidase. Free radicals can be produced by redox and peroxide of transition metal ions (iron and copper). Therefore, promoting antioxidant enzyme production as well as reducing oxidase and metal ion formation can achieve a good antioxidant effect [1].

Sea buckthorn fruits have the highest antioxidant activity among medicinal plants [30]. The active compounds in sea buckthorn that are responsible the plant’s antioxidant properties are resumed in Table 5.

Gallic acid, one of the dominant phenols in sea buckthorn, has been reported to be the most effective antioxidant [86]. Taken together, sea buckthorn seed oil, leaf, branches, and root extracts have significant potential as natural antioxidants and could be used potentially for food additives and the development of useful natural compounds [87]. Several studies revealed the fact that the antioxidant activity of seed and root sea buckthorn extracts is better than that of leaf and stem extracts, using ABTS (2,2′-azino-bis(3-ethylbenzothiazoline-6-sulfonic acid) diammonium salt), DPPH (1,1-diphenyl-2-picrylhydrazyl), and FRAP (ferric reducing antioxidant power) assays [76,85]. 

Su et al. [88] studied the antioxidant capacity of ethanol extracts from sea buckthorn berries by means of ABTS radical-scavenging activity assay, in vitro. The results showed that the ethanol extract of sea buckthorn had a good scavenging effect on ABTS free radicals [88]. Additionally, Rop et al. [89] studied the total contents of phenols and flavonoids as well as antioxidant capacity and scavenging activity of methanolic extracts on reactive oxygen species and lipid peroxidation in some cultivars of sea buckthorn suitable for growing in Central Europe. Altogether, six cultivars of *Hippophaë rhamnoides* L. presented total phenolic contents ranging from 8.62 g of gallic acid/kg fresh weight to 14.17 g of gallic acid/kg fresh weight, the flavonoids content ranged from 4.18 g of rutin/ kg fresh weight to 7.97 g of rutin/kg fresh weight, the ascorbic acid content ranged from 3.94 g/kg fresh weight to 5.73 g/kg fresh weight and the antioxidant capacity, measured by means of the DPPH test, ranged between 11.26 g of ascorbic acid equivalent/kg fresh weight and 18.11 g of ascorbic acid equivalent/kg fresh weight [89].

Dietotherapy with antioxidant foods is a very convenient and effective method for the supplementation of endogenous antioxidants to alleviate damage due to free radicals [88]. However, antioxidants in foods do not necessarily protect biological tissues from free radical oxidative damage because they have to be converted into usable forms in tissues and interact with other substances, in addition to effective concentration differences, and they must display difficulty in absorption from the diet [90].

Several in vivo studies of sea buckthorn’s antioxidant properties proved its obvious antioxidant effects as, for example, sea buckthorn seed extract improves the activity of antioxidant enzymes, and thus has an antiaging effect [38], sea buckthorn seed oil has a good iron-chelating effect, and has a certain protective effect against oxidative damage [91], total flavones from sea buckthorn have antioxidant effects and indirectly inhibit retinal cell apoptosis [87] and has a potent inhibitory effect on lipid peroxidation [92].

## 5. Sea Buckthorn Applications

Sea buckthorn has multiple economic advantages, as a raw material for obtaining cosmetics and nutraceutical preparations, but also for environmental protection. Due to its high tolerance to cold, drought, salt and the ability to fix nitrogen in the soil, it has been identified as an ideal crop for soil and water conservation and to form wind barriers in marginal areas prone to erosion [22].

In recent years, researchers in the fields of nutrition, food science, medicine, sports science, agriculture and forestry have performed numerous studies on *Hippophaë* species, supporting its use as a medicine and food [1]. 

### 5.1. Therapeutic Uses of Sea Buckthorn

Sea buckthorn fruits are traditionally known for their medicinal properties as well as for their high nutritional value. Although used for centuries in Europe and Asia, sea buckthorn fruits have recently gained worldwide popularity, mainly for their nutritional and therapeutic value. They are used in about 200 industrial products, including classic and natural medicines prescribed to treat cancer, heart disease, ulcers, liver disorders, burns, and brain disorders, etc. [3,25,32].

The volume of experimental data attesting the important properties of many bioactive ingredients and substances in sea buckthorn is vast and continues to grow rapidly. Numerous studies have been performed describing the health benefits of sea buckthorn fruit products. A summary of them is presented in Table 6.

Considering all available data, it is safe to say that *Hippophaë* is a promising plant, with many compounds with therapeutic functions, which, integrated in the diet, brings important benefits to human health.

### 5.2. Sea Buckthorn in the Cosmetic Industry

Cosmetic products made with and from sea buckthorn extracts are produced worldwide. These include beauty care products derived from *H. rhamnoides* L., such as moisturizing and facial creams, facial masks, hair lotions, hygiene products such as shampoo, skin cream and bath soak and even oral hygiene products such as sea buckthorn-based mouthwash [117,118].

### 5.3. Sea Buckthorn in the Food Industry

In addition to the different areas of importance of all the component parts of the plant, but also of the sea buckthorn bush, as a whole, the most important sector of use of sea buckthorn remains the food industry, where it is used as a raw material for obtaining functional foods or food supplements. Fruits are the main valuable component in this regard, although the leaves are also used to make sea buckthorn tea. The most important parts of sea buckthorn fruit, of interest in the food industry, are the pulp of the fruit (for obtaining sea buckthorn juice) and the seed of the fruit (for extracting oil) [24,55].

There is a wide range of products obtained from sea buckthorn fruit, as wide as the assortments of generally available products obtained from any other fruit [2]. As of 2018, there have been more than 200 kinds of products derived from *Hippophaë* species [1].

#### 5.3.1. Food Supplements and Food Additives

In the food market, sea buckthorn products with the addition of sea buckthorn oil have an important and well-defined place. Sea buckthorn oils can be found in the composition of certain food supplements, for example those that improve the condition of mucous membranes. These supplements are attractive to consumers because they are a natural source of health benefits [119]. In Finland, sea buckthorn is used as a functional ingredient in baby food [120]. Residues from sea buckthorn juice are a good functional addition for mechanically boned and manually boned meat products, due to the inhibition of the breakdown of fatty acids contained in them and the enrichment of meat with polyphenols. Studies have shown that the addition of 2% sea buckthorn powder does not cause negative changes in the organoleptic characteristics of products made from hand-boned meat (taste, smell and texture) [121]. “Sea buckthorn yellow” can be extracted from the peel left after the juice is removed, a pigment that can be used as a food coloring additive [24]. A *H. rhamnoides* L. fruit peel powder, which is rich in antioxidant nutrients, is used as a nutritional supplement that can significantly enhance the body’s free radical-scavenging function and improve antioxidant function in immunocompromised people or people with antioxidant hypofunction [1]. Another commercially available sea buckthorn product, additive free and with unique taste, is a health-promoting edible salt product of *H. rhamnoides* L. that has high nutritional value and exerts excellent antioxidant effects [1].

#### 5.3.2. Refreshments

Some of the most popular and oldest sea buckthorn products are juices and refreshing drinks. These nutritious drinks, rich in vitamin C and carotene are very popular in China, Germany, Scandinavia, and other Nordic countries. At the 1992 Seoul Olympics, sea buckthorn refreshing drinks were the official drinks of Chinese athletes [19]. They were also used in the diet of Indian soldiers when they were working at very low temperatures [122]. 

#### 5.3.3. Jams and Jellies

Despite the sour and exotic taste, sea buckthorn berries can be used for the production of jams and jellies. Its intense aroma can be neutralized by mixing juice or sea buckthorn paste and other fruits with a much milder taste, in different proportions [30]. Research has shown that sea buckthorn fruit, due to its high content of biologically active compounds, is a valuable fruit for the production of jams. In the study presented by Rafalska et al. [114], a variety of jams with different flavors and colors were prepared, mixing sea buckthorn fruits with other fruits (apples, currants, raspberries, and strawberries). Sensory analysis showed that sea buckthorn jams with the addition of strawberries and raspberries were the most appreciated by evaluators [114].

#### 5.3.4. Dairy Products

Sea buckthorn has been used as an ingredient in dairy products such as kefir, yogurt, or cheese. Existing studies have shown that the addition of sea buckthorn puree has led to a significant increase in the antioxidant properties of fermented drinking beverages and, in addition, has led to an increase in the acidity of the products tested [114]. Sea buckthorn fruits were also used in the production of feta cheese, in which they formed a biodegradable skeleton on which the beneficial probiotic bacterial strain *Lactobacillus casei* ATCC 393 could develop. In addition, the addition of sea buckthorn also contributed to reduce the number of pathogenic microorganisms and improved the organoleptic properties of cheeses [123].

#### 5.3.5. Alcoholic Beverages

It is possible to produce alcoholic beverages with the use of sea buckthorn [114]. Sea buckthorn tincture prepared from sea buckthorn has long been used as an adjuvant in many diseases of the digestive system, such as slow bowel syndrome. It can improve the functioning of the stomach and maintain the normal activity of the gastrointestinal tract [119]. Furthermore, sea buckthorn wine has gained the greatest popularity in the Czech Republic, where it is widely produced. Sea buckthorn wine is characterized by its golden color and pleasant aroma [19]. Sea buckthorn is also used as raw material in beer production [124]. Besides that, previous work of authors of the present study provided a solution for the capitalization of by-products resulting from processing sea buckthorn in the juice industry, namely the optimization of the fermentation process of sea buckthorn marc in order to obtain a refreshing low alcoholic beverage [125].

## 6. Conclusions

Sea buckthorn (*Hippophaë rhamnoides* L.) is a unique plant, considering its chemical composition and the therapeutic properties that rise from its composition. Not for nothing, sea buckthorn is widely distributed and estimated to cover about 3.0 million hectares worldwide (both wild and cultivated) [126]. Additionally, sea buckthorn has multiple economic advantages, as a raw material for obtaining cosmetics and nutraceuticals, but also for environmental protection [22]. In addition to the different areas of importance of all the component parts of the plant, but also of the sea buckthorn bush, as a whole, the most important sector of use of sea buckthorn remains the food industry, where it is used as a raw material for obtaining functional foods or food supplements, which can be successfully integrated in plant-based diets for consumers who engage in a healthy lifestyle or for those who are interested in preventing or treating diet-related diseases. Fruits are the main valuable component in this regard, but leaves or by-products resulting from processing the fruits can also be capitalized.

The phytochemical and nutritional composition of sea buckthorn berries, hence their nutritional value, differs considerably depending on the species, components analyzed, climatic and growing conditions, variations between years, degree of maturation, storage conditions, time of harvest, and method of processing and analysis [23].

The most valuable component of sea buckthorn fruits is their oil. Sea buckthorn seed oil is characterized by a high content of oleic acid (17%) and a one-to-one ratio of omega-3 (alpha linolenic) and omega-6 (linoleic). The equivalence relationship between the two omegas is particularly important, intervening in the regulation of thousands of metabolic functions. Almost every biological function is interconnected with the balance between omega-6 and omega-3 [30,32]. Additionally, sea buckthorn is a very good source of phytosterols, which play an important role in prophylaxis of cardiovascular diseases induced by hypercholesterolemia [3]. 

Another important health-promoting aspect of sea buckthorn is its high fiber content and the fact that it is considered a unique protein source [3]. Sea buckthorn juice is rich in many free amino acids, too. A total of 18 of the 22 known amino acids have been found in sea buckthorn fruits, half of which are essential because they play a critical role in various processes in the human body [47,48]. However, sea buckthorn is not that valuable when considering its content in mineral elements.

The main substances present in large quantities in the pulp of sea buckthorn fruits belong to the carotenoids class, the carotenoid content being one of the key characteristics by which sea buckthorn oil is traded commercially [2]. Yet, the most important therapeutic element in sea buckthorn fruits is the ascorbic acid [23,65]. An important feature, differentiating *Hippophaë* from other fruits, is that sea buckthorn berries do not contain ascorbate oxidase, the enzyme responsible for the degradation of ascorbic acid and therefore, sea buckthorn products and even dried fruits still contain large amounts of vitamin C [67].

*Hippophaë* fruits have a unique flavor that cannot be compared to the flavor of any other fruit. Sea buckthorn fruits are known to have a specific scent based on around 45 compounds, its description considering notes of berry or citrus and even pineapple [19]. The taste of sea buckthorn juice was characterized by astringency, sourness, and bitterness [42]. Further studies should engage in a more detailed characterization of sea buckthorn sensory properties, as there are very few available researches in which the aroma compounds that are responsible for the sensory properties of sea buckhorn juices are studied.

The volume of data stating the importance of sea buckthorn as a plant with great chemical composition and therapeutic value is vast and continues to grow rapidly. This review is a detailed picture of the current state of knowledge currently available regarding this unique plant, cultivated and appreciated around the globe, providing an overview of the qualities of sea buckthorn fruits derived from their exceptional chemical composition. Therefore, *Hippophaë* is a valuable plant, with many biologically active compounds with therapeutic functions, which, integrated into the diet, can bring important benefits to human health.

## Figures and Tables

**Table 1:** Physico-chemical properties of sea buckthorn juice/berries [2,23,24,25,26].

Characteristic (U.M.)	Range of Variation of the Values	Mean Value	Species/Variety
Fruit weight (mg)	270–480	350	*Indian Summer* subsp.
Moisture (%)	73.60–85.30	82.30	*Indian Summer* subsp.
	72.20–75.50	74.20	China var.
pH	2.50–2.73	2.65	*Carpatica* subsp.
Acidity (% malic acid)	1.64–1.74	1.69	*Carpatica* subsp.
Juice oil content (%)	0.26–1.43	0.90	China var.
	1.80–2.90 (pulp)	2.00	China var.
Unit weight	1.03–1.05	1.04	China var.
Conductivity (μΩ/m)	0.30–0.54	0.36	China var.
Surface tension (N/m)	46.23–55.14	50.74	China var.
Refractive index	1.35–1.36	1.35	China var.
Soluble sugars (°Brix)	9.30–17.30	11.40	*Indian Summer* subsp.
	10.19–22.74	15.98	China var.
	6.40–12.70 (reducing sugars)	9.00	China var.
Crude protein (g/kg dry weight)	86–100	93	Poland var.
Crude fiber (g/kg dry weight)	62–73	67.5	Poland var.
Ash (g/kg dry weight)	40–41	40.5	Poland var.

**Table 2:** Phytosterols content in sea buckthorn pulp and seed oil, (g/kg of fresh weight).

Phytosterol	Pulp Oil	Seed Oil	Reference
β-sitosterol	5.2–5.7	5.9–7.9	[2,10]
		9.9	[38]
		11.8	[15]
Δ5-Avenasterol		3.3	[38]
Cycloartenol		1.9	[38]
Stigmasterol	1.0–1.2	1.2	[15]
Gramisterol		0.6	[38]
Citrostadienol		0.5	[15]
Campesterol		0.3	[15,38]
Total sterols	20–30	10–20	[10]

**Table 3:** Content of various amino acids in sea buckthorn juice.

Amino Acid	Content (mg/100 g)	Variety	Reference
Aspartic acid/Asparagine	426.6	*H. rhamnoides* L.	[50]
	3.7	*H. rhamnoides* subsp. *sinensis*	[51]
	427	-	[10]
Proline	45.2	*H. rhamnoides* L.	[50]
	12.3	*H. rhamnoides* subsp. *sinensis*	[51]
	45	-	[10]
Threonine	36.8	*H. rhamnoides* L.	[50]
	6.2	*H. rhamnoides* subsp. *sinensis*	[51]
	37	-	[10]
Serine	28.1	*H. rhamnoides* L.	[50]
	5.3	*H. rhamnoides* subsp. *sinensis*	[51]
	28	-	[10]
Lysine	27.2	*H. rhamnoides* L.	[50]
	3.5	*H. rhamnoides* subsp. *sinensis*	[51]
Valine	21.8	*H. rhamnoides* L.	[50]
	2.9	*H. rhamnoides* subsp. *sinensis*	[51]
Alanine	21.2	*H. rhamnoides* L.	[50]
	2.5	*H. rhamnoides* subsp. *sinensis*	[51]
Phenylalanine	20	*H. rhamnoides* L.	[50]
	3.2	*H. rhamnoides* subsp. *sinensis*	[51]
Glutamine	19.4	*H. rhamnoides* L.	[50]
	2.7	*H. rhamnoides* subsp. *sinensis*	[51]
Isoleucine	17.4	*H. rhamnoides* L.	[50]
	1	*H. rhamnoides* subsp. *sinensis*	[51]
Glycine	16.7	*H. rhamnoides* L.	[50]
	0.6	*H. rhamnoides* subsp. *sinensis*	[51]
Histidine	13.7	*H. rhamnoides* L.	[50]
	1.1	*H. rhamnoides* subsp. *sinensis*	[51]
Tyrosine	13.4	*H. rhamnoides* L.	[50]
	1.8	*H. rhamnoides* subsp. *sinensis*	[51]
Arginine	11.3	*H. rhamnoides*. L.	[50]
	0.5	*H. rhamnoides* subsp. *sinensis*	[51]
Cysteine	3.3	*H. rhamnoides* L.	[50]
	0.8	*H. rhamnoides* subsp. *sinensis*	[51]
Methionine	2.3	*H. rhamnoides* L.	[50]
	1.1	*H. rhamnoides* subsp. *sinensis*	[51]
Leucine	1.9	*H. rhamnoides* subsp. *sinensis*	[51]
Tryptophan	0.5	*H. rhamnoides* subsp. *sinensis*	[51]
Essential	155.9	*H. rhamnoides* L.	[50]
	21.6	*H. rhamnoides* subsp. *sinensis*	[51]
Total	724.4	*H. rhamnoides* L.	[50]
	51.6	*H. rhamnoides* subsp. *sinensis*	[51]

**Table 4:** The mineral composition of sea buckthorn fresh berries.

Mineral Element	Content Variation Range or Median (mg/kg)	Origin of the Variety	Reference
K	100–806	Chinese	[53]
	590–2070	Not indicated	[51]
	636–1192	Turkish	[50]
	3020	German	[54]
	3790	Romanian	[54]
Ca	64–256	Chinese	[53]
	120–720	Not indicated	[51]
	126–547	Turkish	[50]
	46.70	German	[54]
	47.50	Romanian	[54]
P	58–95	Not indicated	[51]
	610–990	Turkish	[50]
Mg	53.30–165	Chinese	[53]
	300	Not indicated	[51]
	187–190	Turkish	[50]
	123	German	[54]
	85.50	Romanian	[54]
Na	18–89.80	Chinese	[53]
	10–40	Not indicated	[51]
	172–208	Turkish	[50]
	12.50	German	[54]
	20.60	Romanian	[54]
Co	<0.10	Chinese	[48]
	0.01–0.09	Chinese	[53]
Cr	0.11–0.29	Chinese	[48]
	0.47–1.00	Chinese	[53]
Cu	0.16–0.65	Chinese	[48]
	2.30–3.40	Turkish	[50]
	1.01	German	[54]
	0.99	Romanian	[54]
Mn	1.17–2.60	Chinese	[48]
	0.81–3.86	Chinese	[53]
	2.70–4.00	Turkish	[50]
	3.20	German	[54]
	2.76	Romanian	[54]
Ni	0.12–0.36	Chinese	[48]
	0.39–0.09	Chinese	[53]
	0.38	German	[54]
	0.62	Romanian	[54]
Sr	0.19–0.62	Chinese	[48]
	0.08–0.45	Chinese	[53]
V	0.002–0.01	Chinese	[48]
Fe	4.13–10.90	Chinese	[48]
	5.93–161	Chinese	[53]
	4.40	Not indicated	[51]
	0.46–1.27	Turkish	[50]
	3.70	German	[54]
	2.47	Romanian	[54]
Mo	0.03–0.06	Chinese	[48]
	1.18	Chinese	[53]
	0.15	German	[54]
	0.083	Romanian	[54]
Zn	0.43–1.25	Chinese	[48]
	2.09–6.31	Chinese	[53]
	3.00–3.90	Turkish	[50]
	1.78	German	[54]
	2.24	Romanian	[54]
Sn	0.05–0.26	Chinese	[48]
Se	7.96–11.30	Chinese	[48]
B	0.43–1.38	Chinese	[48]
	2.90	German	[54]
Ba	0.17–0.36	Chinese	[48]
Al	2.20–16.70	Chinese	[48]
Ti	0.10–0.81	Chinese	[48]
Li	0.13–0.30	Chinese	[48]
	0.06–0.15	Chinese	[53]
Cd	<0.05	Chinese	[48]
	0.002–0.015	Chinese	[53]
As	<0.5	Romanian	[56]
Pb	0.43–0.76	Chinese	[48]
	0.06–0.27	Chinese	[53]

**Table 5:** Antioxidant compounds in sea buckthorn.

Part of the Plant	Antioxidant Compounds	Reference
Berries	Carotenoids, flavonoids, and organic acids	[79]
Seeds	Catechin, epicatechin, gallocatechin, and epigallocatechin	[83]
	Catechin(4α-8)catechin and catechin(4α-8)epicatechin	[84]
Oil	Carotenoids and β-sitosterol	[55]
	Monounsaturated fatty acids	[4]
	Tocopherols and tocotrienols	[33]
Leaves	Kaempferol-3-O-β-D-(6″-O-coumaryl)glycoside, 1-feruloyl-β-D-glucopyranoside, isorhamnetin-3-O-glucoside, quercetin-3-O-β-D-glucopyranoside, quercetin-3-O-β-Dglucopyranosyl-7-O-α-L-rhamnopyranoside and isorhamnetin-3-O-rutinoside	[85]

**Table 6:** Medicinal properties of sea buckthorn (*Hippophaë rhamnoides* L.).

Medicinal Property	Sea Buckthorn Type	Place of Origin	Reference
Anticarcinogenic	*Hippophae rhamnoides* (L.)	Uchacg, France	[76]
		Lund, Sweden	[93]
		Québec, Canada	[94]
Antimutagenic	*Hippophae rhamnoides* (L.)	Ulan-Ude, Siberia	[95]
Antitumor	*Hippophae rhamnoides* (L.)	Tianjin, China	[96]
Immunomodulating	*Hippophae rhamnoides* (L.)	Western Himalayas, India	[79]
		Sokołka, Poland	[97]
Radiation protection	*Hippophae rhamnoides* (L.)	Himachal Pradesh, India	[98]
Cardiovascular disease	*Hippophae rhamnoides* (L.)	Ladakh, India	[99]
Antibacterial, antiviral	*Hippophae rhamnoides* (L.)	Kaza, India	[100]
Antioxidative and weight loss	*Hippophae rhamnoides* ssp. *turkestanica*	Turku, Finland	[101]
Arterial thrombosis	*Hippophae rhamnoides* (L.)	Shenyang, China	[69]
Antiatherogenic	*Hippophae rhamnoides* (L.)	Western Himalayas, India	[102]
Gastric ulcer	*Hippophae rhamnoides* ssp. *rhamnoides*	Romania	[18]
	*Hippophae rhamnoides* (L.)	Tortum, Turkey	[103]
Infections of the digestive tract	*Hippophae rhamnoides* spp. *mongolica*	Ostrobothnia, Finland	[104]
Hepatic fibrosis	Granules of sea buckthorn extract	Sichuan Pharmaceutical Co., Ltd., China	[105]
Dermatological conditions	*Hippophae rhamnoides* (L.)	Skardu, Pakistan	[106]
	*Hippophae rhamnoides* (L.) (sea buckthorn oil)	Dongning Pharmceutical Co., Ltd., China	[107]
Common cold	*Hippophae rhamnoides* (L.)	Olsztyn, Poland and Belorussia	[64,104]
	*Hippophae rhamnoides* spp. *mongolica*	Ostrobothnia, Finland	[104]
			
Ophthalmic conditions	*Hippophae rhamnoides* (L.) (oil capsules)	Aromtech Ltd., Finland	[108]
Chronic vaginal inflammation	*Hippophae rhamnoides* (L.) (oil capsules)	Aromtech Ltd., Finland	[109,110]
Healing effect on acute and chronic wounds	*Hippophae rhamnoides* ssp. *rhamnoides*	Romania	[18]
	*Hippophae rhamnoides* (L.)	Kazeroon, Iran	[111,112]
	*Hippophae rhamnoides* (L.)	Western Himalayas, India	[113]
Anti-inflammatory	*Hippophae rhamnoides* (L.)	Olsztyn, Poland and Belorussia	[114]
Antidiabetic	*Hippophae rhamnoides* (L.)	Chifeng, Inner Mongolia	[115]
	*Hippophae rhamnoides* (L.)	New Delhi, India	[116]

## Data Availability

No new data were created or analyzed in this study. Data sharing is not applicable to this article.
